# Supplementary material for: Minigene as a Novel Regulatory Element in Toxin-Antitoxin Systems
Source: Int J Mol Sci. 2021 Dec 13;22(24):13389. doi: 10.3390/ijms222413389 (PMC8708949; doi:10.3390/ijms222413389)
Supplement: Supplementary file 1 [file ijms-22-13389-s001.zip › ijms-1489209-supplementary.pdf]

Minigene as a novel regulatory element in the toxin-antitoxin systems

Barbara Kędzierska [1,*], Katarzyna Potrykus [1]

Table S1. Plasmids used in this work.

Plasmid	Description	Source
pRW225	RK2-based low copy number vector; enables cloning promoter fragment carrying own translation signals in fusion to lacZ gene between EcoRI and HindIII; Tet^R	[24]
pRW_ATGmg1	A derivative of pRW225 with paxe promoter sequence up to the ATG 1 amplified with primers 29 and 141; ATG acts as a start codon for lacZ reporter gene.	This work
pRW_GTG	A derivative of pRW225 with paxe promoter sequence up to the GTG amplified with primers 29 and 203; GTG acts as a start codon for lacZ reporter gene.	This work
pRW_ATGmg2	A derivative of pRW225 with paxe promoter sequence up to the ATG 2 amplified with primers 29 and 142; ATG acts as a start codon for lacZ reporter gene.	This work
pRW_ATGtxe	A derivative of pRW225 with paxe promoter sequence up to the ATG txe amplified with primers 29 and 143; ATG acts as a start codon for lacZ reporter gene.	This work
pRW_ATGtxe_ATGmg1mut	A derivative of pRW_ATGtxe where ATG 1 was mutated to ACG with primers 174 and 175	This work
pRW_ATGtxe_GTG/SDmut	A derivative of pRW_ATGtxe where GTG was mutated to CTG with primers 204 and 205	This work
pRW_ATGtxe_ATGmg2mut	A derivative of pRW_ATGtxe where ATG 2 was mutated to ACG with primers 98 and 140	This work
pRW_ATGtxe_mg2mut	A derivative of pRW_ATGtxe where ATG and TAA of mg2 were mutated to ACG and TTA with primers 178 and 179	This work
pRW_ATGtxe_mg1,2mut	A derivative of pRW_ATGtxe_mg2mut where ATG 1 was mutated to ACG with primers 174 and 175	This work
pRW_ATGtxe_w/o mg1,2	A derivative of pRW_ATGtxe where a sequence with minigenes was deleted with primers 143 and 211	This work
pRW_ATGtxe-1	A derivative of pRW_ATGtxe where the distance between ATG 2 and ATG txe was changed by -1 with primers 143 and 212 (deletion of T)	This work
pRW_ATGtxe-2	A derivative of pRW_ATGtxe where the distance between ATG 2 and ATG txe was changed by -2 with primers 143 and 214 (deletion of TC)	This work
pRW_ATGtxe+1	A derivative of pRW_ATGtxe where the distance between ATG 2 and ATG txe was changed by +1 with primers 143 and 207 (insertion of C)	This work
pRW_ATGtxe+2	A derivative of pRW_ATGtxe where the distance between ATG 2 and ATG txe was changed by +2 with primers 143 and 209 (insertion of TC)	This work
pRW_ATGtxe+3	A derivative of pRW_ATGtxe where the distance between ATG 2 and ATG txe was changed by +3 with	This work

	primers 143 and 216 (insertion of CTC)	
pRW_ATGtxe+4	A derivative of pRW_ATGtxe where the distance between ATG 2 and ATG txe was changed by +4 with primers 143 and 218 (insertion of TCTC)	This work
pRW_ATGtxe+5	A derivative of pRW_ATGtxe where the distance between ATG 2 and ATG txe was changed by +5 with primers 143 and 220 (insertion of CTCTC)	This work
pRW_ATGtxe+6	A derivative of pRW_ATGtxe where the distance between ATG 2 and ATG txe was changed by +6 with primers 143 and 222 (insertion of TCTCTC)	This work
pRW_ATGtxe-1_mg2mut	A derivative of pRW_ATGtxe-1 with the mg2 mutated with primers 178 and 179	This work
pRW_ATGtxe-2mg2mut	A derivative of pRW_ATGtxe-2 with the mg2 mutated with primers 178 and 179	This work
pRW_ATGtxe+1_mg2mut	A derivative of pRW_ATGtxe+1 with the mg2 mutated with primers 178 and 179	This work
pRW_ATGtxe+2_mg2mut	A derivative of pRW_ATGtxe+2 with the mg2 mutated with primers 178 and 179	This work
pRW_ATGtxe+3_mg2mut	A derivative of pRW_ATGtxe+3 with the mg2 mutated with primers 178 and 179	This work
pRW_ATGtxe+4_mg2mut	A derivative of pRW_ATGtxe+4 with the mg2 mutated with primers 178 and 179	This work
pRW_ATGtxe+5_mg2mut	A derivative of pRW_ATGtxe+5 with the mg2 mutated with primers 178 and 179	This work
pRW_ATGtxe+6_mg2mut	A derivative of pRW_ATGtxe+6 with the mg2 mutated with primers 178 and 179	This work
pRW_ATGtxe-1_mg2mut_mg1mut	A derivative of pRW_ATGtxe-1_mg2mut with the mg1 mutated with primers 174 and 175	This work
pRW_ATGtxe+2_mg2mut_mg1mut	A derivative of pRW_ATGtxe+2_mg2mut with the mg1 mutated with primers 174 and 175	This work
pRW_ATGtxe-1_mg2mut_GTGmut	A derivative of pRW_ATGtxe-1_mg2mut with the GTG mutated with primers 204 and 205	This work
pRW_ATGtxe+2_mg2mut_GTGmut	A derivative of pRW_ATGtxe+2_mg2mut with the GTG mutated with primers 204 and 205	This work
pBAD33	Arabinose-inducible expression vector, Cm^R	[47]
pBAD33mg2	A derivative of pBAD33 with the fragment containing minigene 2 amplified with primers 143 and 198, inserted under control of p_{BAD} promoter between PstI and HindIII restriction sites	This work
pBAD33mg1,2	A derivative of pBAD33 with the fragment containing minigenes 1 and 2 amplified with primers 143 and 197, inserted under control of p_{BAD} promoter between PstI and HindIII restriction sites	This work

Table S2. Oligonucleotides used in this study. Restriction enzyme sites and mutated positions are underlined.

Name	Sequence (5′-3′)	Description
29	CGCGGGAATTCTAGAAATAAATAAGGGGT	Forward primer containing EcoRI site for amplification of paxe promoter fragments to clone to pRW225
141	GCAAAGCTTCATCTCCTCGACG	Reverse primer containing HindIII site for amplification of a fragment with ATG mg1 fused to lacZ to clone to pRW225
203	GCAAAGCTTCACCTTTGGAGAATTG	Reverse primer containing HindIII site for amplification of a fragment with GTG fused to lacZ to clone to pRW225
142	GCAAAGCTTCATGTGTTTTAAATGC	Reverse primer containing HindIII site for amplification of a fragment with ATG mg2 fused to lacZ to clone to pRW225
143	GCAAAGCTTCATCAGATTCAACCTCG	Reverse primer containing HindIII site for amplification of a fragment with ATG txe fused to lacZ to clone to pRW225
174	GTCGAGGAGACGAACAATTCTC	Forward primer for mutagenesis of ATG mg1 (ATG→ACG)
175	GAGAATTGTTCGTCTCCTCGAC	Reverse primer for mutagenesis of ATG mg1 (ATG→ACG)
204	CTCCAAAGCTGCATTTAAAC	Forward primer for mutagenesis of GTG/SD (GTG→CTG)
205	GTTTAAATGCAGCTTTGGAG	Reverse primer for mutagenesis of GTG/SD (GTG→CTG)
98	CATTTAAAACACACGACTTAATC	Forward primer for mutagenesis of ATG mg2 (ATG→ACG)
140	GATTAAGTCGTGTGTTTTAAATG	Reverse primer for mutagenesis of ATG mg2 (ATG→ACG)
179	ATTTAAAACACACGACCTTATCGAGGTTG	Forward primer for mutagenesis of ATG and STOP codons of mg2 (ATG→ACG and TAA→TTA)
178	CAACCTCGATAAGGTCGTGTGTTTTAAAT	Reverse primer for mutagenesis of ATG and STOP codons of mg2 (ATG→ACG and TAA→TTA)
211	GCAAAGCTTCATCAGATTCAACCTCGAAAGTGTTCTCAACG	Reverse primer for amplification of paxe promoter fragment to the ATG txe, devoid of minigenes sequence in between
212	GCAAAGCTTCATCAGATTCAACCTCGTTAAGTCATG	Reverse primer for amplification of paxe fragment to ATG txe with a deletion of T
214	GCAAAGCTTCATCAGATTCAACCTCTTAAGTCATG	Reverse primer for amplification of paxe fragment to ATG txe with a deletion of TC
207	GCAAAGCTTCATCAGATTCAACCTCGAGTTAAGTCATG	Reverse primer for amplification of paxe fragment to ATG txe with an insertion of C
209	GCAAAGCTTCATCAGATTCAACCTCGAGATTAAGTCATG	Reverse primer for amplification of paxe fragment to ATG txe with an insertion of TC
216	GCAAAGCTTCATCAGATTCAACCTCGAGAGTTAAGTCATG	Reverse primer for amplification of paxe fragment to ATG txe with an insertion of CTC

218	GCAAAGCTTCATCAGATTCAACCTCGAGAGATTAAGTCATG	Reverse primer for amplification of paxe fragment to ATG txe with an insertion of TCTC
220	GCAAAGCTTCATCAGATTCAACCTCGAGAGAGTTAAGTCATG	Reverse primer for amplification of paxe fragment to ATG txe with an insertion of CTCTC
222	GCAAAGCTTCATCAGATTCAACCTCGAGAGAGATTAAGTCATG	Reverse primer for amplification of paxe fragment to ATG txe with an insertion of TCTCTC
213	GCAAAGCTTCATCAGATTCAACCTCGTAAGGTCGTGTG	Reverse primer for amplification of paxe fragment to ATG txe with a deletion of T and mutation of mg2
215	GCAAAGCTTCATCAGATTCAACCTCTAAGGTCGTGTG	Reverse primer for amplification of paxe fragment to ATG txe with a deletion of TC and mutation of mg2
208	GCAAAGCTTCATCAGATTCAACCTCGAGTAAGGTCGTGTG	Reverse primer for amplification of paxe fragment to ATG txe with an insertion of C and mutation of mg2
210	GCAAAGCTTCATCAGATTCAACCTCGAGATAAGGTCGTGTG	Reverse primer for amplification of paxe fragment to ATG txe with an insertion of TC and mutation of mg2
217	GCAAAGCTTCATCAGATTCAACCTCGAGAGTAAGGTCGTGTG	Reverse primer for amplification of paxe fragment to ATG txe with an insertion of CTC and mutation of mg2
219	GCAAAGCTTCATCAGATTCAACCTCGAGAGATAAGGTCGTGTG	Reverse primer for amplification of paxe fragment to ATG txe with an insertion of TCTC and mutation of mg2
221	GCAAAGCTTCATCAGATTCAACCTCGAGAGAGTAAGGTCGTGTG	Reverse primer for amplification of paxe fragment to ATG txe with an insertion of CTCTC and mutation of mg2
223	GCAAAGCTTCATCAGATTCAACCTCGAGAGAGATAAGGTCGTGTG	Reverse primer for amplification of paxe fragment to ATG txe with an insertion of TCTCTC and mutation of mg2
197	TAGTCTGCAGTGGAAAAAATTCGTCGAGG	Forward primer for amplification of a fragment containing minigenes 1 and 2 sequence, to clone to pBAD33, with PstI site
198	TAGTCTGCAGTGAACAATTCTCCAAAGGTGC	Forward primer for amplification of a fragment containing minigene 2 sequence, to clone to pBAD33, with PstI site

Table S3. A list of bacterial strains tested for the presence of two-ORF minigenes. In the indicated TA families two-ORF minigenes were not found.

Bacterial strain	TA family
Escherichia coli CFT073	*yefM-yoeB*
Escherichia coli str. K-12 substr. MG1655	*yefM-yoeB*
Lactobacillus rhamnosus Lc 705	*yefM-yoeB*
Streptomyces avermitilis MA-4680	*yefM-yoeB*
Streptomyces coelicolor A3(2)	*yefM-yoeB*
Agrobacterium tumefaciens str. C58 Atu0935	*relBE*
Agrobacterium tumefaciens str. C58 Atu0675	*relBE*
Agrobacterium vitis S4	*relBE*
Aliivibrio salmonicida LFI1238	*relBE*
Archaeoglobus fulgidus DSM 4304 AF1094	*relBE*
Archaeoglobus fulgidus DSM 4304 AF1081	*relBE*
Archaeoglobus fulgidus DSM 4304 AF1076	*relBE*
Bacteroides fragilis YCH46	*relBE*
Bartonella henselae str. Houston-1 BH07070	*relBE*
Bifidobacterium longum subsp. infantis ATCC 15697	*relBE*
Bradyrhizobium diazoefficiens USDA 110	*relBE*
Brucella melitensis str. 16M	*relBE*
Brucella suis 1330	*relBE*
Campylobacter jejuni RM1221	*relBE*
Candidatus Protochlamydia amoebophila UWE25 pc0996	*relBE*
Candidatus Protochlamydia amoebophila UWE25 pc1913	*relBE*
Caulobacter crescentus CB15 CC_2514	*relBE*
Caulobacter crescentus CB15 CC_0803	*relBE*
Desulfovibrio vulgaris str. Hildenborough	*relBE*
Geobacillus kaustophilus HTA426	*relBE*
Geobacter sulfurreducens PCA GSU2438	*relBE*
Gloeobacter violaceus PCC 7421 glr4239	*relBE*
Gloeobacter violaceus PCC 7421 gsr3527	*relBE*
Gloeobacter violaceus PCC 7421 gsl1825	*relBE*
Helicobacter pylori 26695 HP0895	*relBE*
Idiomarina loihiensis L2TR IL1162	*relBE*
Legionella pneumophila str. Lens lpl1587	*relBE*
Methanococcus maripaludis S2 MMP0477	*relBE*
Methanosarcina mazei Go1 MM_2681	*relBE*
Methylococcus capsulatus str. Bath MCA2652	*relBE*
Mycobacterium bovis AF2122/97 Mb3392	*relBE*
Mycobacterium tuberculosis CDC1551 MT3465	*relBE*
Mycobacterium tuberculosis H37Rv Rv3357	*relBE*
Nitrosomonas europaea ATCC 19718 NE1998	*relBE*
Nitrosomonas europaea ATCC 19718 NE1583	*relBE*
Nitrosomonas europaea ATCC 19718 NE0712	*relBE*
Nostoc sp. PCC 7120 (*Anabaena sp.* PCC 7120) all4408	*relBE*
Pectobacterium atrosepticum SCRI1043 ECA0444	*relBE*
Pectobacterium atrosepticum SCRI1043 ECA0325	*relBE*
Photobacterium profundum SS9 PBPRB1200	*relBE*
Photobacterium profundum SS9 PBPRB0955	*relBE*
Pseudomonas aeruginosa PA1 PA1S_26100	*relBE*
Pseudomonas aeruginosa PA1 PA1S_26070	*relBE*

Pseudomonas aeruginosa PAO1 PA0125	*relBE*
Pseudomonas putida KT2440 PP_2499	*relBE*
Pseudomonas putida KT2440 PP_1268	*relBE*
Pseudomonas syringae pv. Tomato str. DC3000 PSPTO_5388	*relBE*
Pseudomonas syringae pv. Tomato str. DC3000 PSPTO_3676	*relBE*
Shewanella oneidensis MR-1 SO_4642	*relBE*
Sinorhizobium meliloti 1021 SMc00693	*relBE*
Streptococcus agalactiae NEM316 gbs0471	*relBE*
Streptococcus mutans UA159 SMU_895	*relBE*
Streptococcus pneumoniae R6 spr1104	*relBE*
Streptococcus pneumoniae R6 spr0252	*relBE*
Streptococcus pneumoniae TIGR4 SP_0275	*relBE*
Streptomyces cattleya NRRL 8057 = DSM 46488 SCATT_39270	*relBE*
Sulfolobus tokodaii str. 7 STS035	*relBE*
Synechocystis sp. PCC 6803 SYNGTS_2983	*relBE*
Synechocystis sp. PCC 6803 ssr1114	*relBE*
Thauera sp. MZ1T Tmz1t_3767	*relBE*
Thermoanaerobacter tengcongensis MB4	*relBE*
Thermococcus kodakarensis KOD1 TK0966	*relBE*
Thermococcus kodakarensis KOD1 TK0792	*relBE*
Treponema denticola ATCC 35405 TDE1979	*relBE*
Vibrio cholerae O1 biovar El Tor str. N16961 VCA0349	*relBE*
Vibrio cholerae O1 biovar El Tor str. N16961 VCA0504	*relBE*
Clostridium acetobutylicum ATCC 824 CA_C0493	*mazEF*
Deinococcus radiodurans R1 DR_0661	*mazEF*
Deinococcus radiodurans R1 DR_0416	*mazEF*
Desulfovibrio vulgaris str. Hildenborough DVU1510	*mazEF*
Enterococcus faecalis V583 EF3261	*mazEF*
Escherichia coli O157:H7 str. EDL933 Z5835	*mazEF*
Escherichia coli O157:H7 str. Sakai ECs5202	*mazEF*
Geobacter uraniireducens Rf4 Gura_2715	*mazEF*
Gloeobacter violaceus PCC 7421 gsr4049	*mazEF*
Gloeobacter violaceus PCC 7421 gsr2287	*mazEF*
Gloeobacter violaceus PCC 7421 gsr0095	*mazEF*
Lactobacillus johnsonii NCC 533 LJ0588	*mazEF*
Leptospira interrogans serovar Copenhageni str. Fiocruz L1 - 130 LIC11192	*mazEF*
Leptospira interrogans serovar Lai str. 56601 LA_2844	*mazEF*
Leptospira interrogans serovar Lai str. 56601 LA_1780	*mazEF*
Methylococcus capsulatus str. Bath MCA3006	*mazEF*
Mycobacterium bovis AF2122/97 Mb1531	*mazEF*
Mycobacterium bovis AF2122/97 Mb0679c	*mazEF*
Mycobacterium bovis AF2122/97 Mb1978c	*mazEF*
Mycobacterium tuberculosis CDC1551 MT2047	*mazEF*
Mycobacterium tuberculosis CDC1551 MT1541	*mazEF*
Mycobacterium tuberculosis CDC1551 MT0689	*mazEF*
Mycobacterium tuberculosis H37Rv Rv0456B	*mazEF*
Mycobacterium tuberculosis H37Rv Rv1943c	*mazEF*
Mycobacterium tuberculosis H37Rv Rv1494	*mazEF*
Neisseria meningitidis MC58 NMB2037	*mazEF*
Nitrosomonas europaea ATCC 19718 NE1580	*mazEF*

Nocardia farcinica IFM 10152 nfa7700	*mazEF*
Nostoc sp. PCC 7120 (*Anabaena sp*. PCC 7120) asl3212	*mazEF*
Nostoc sp. PCC 7120 (*Anabaena sp*. PCC 7120) asr0757	*mazEF*
Pectobacterium atrosepticum SCRI1043 ECA0583	*mazEF*
Photorhabdus luminescens subsp. laumondii TTO1 plu2366	*mazEF*
Pseudomonas putida KT2440 PP_0770	*mazEF*
Rhodopseudomonas palustris CGA009 RPA0942	*mazEF*
Staphylococcus aureus subsp. aureus COL SACOL2059	*mazEF*
Staphylococcus aureus subsp. aureus MRSA252 SAR2157	*mazEF*
Staphylococcus aureus subsp. aureus MSSA476 SAS1974	*mazEF*
Staphylococcus aureus subsp. aureus Mu50 SAV2069	*mazEF*
Staphylococcus aureus subsp. aureus MW2 MW1993	*mazEF*
Staphylococcus aureus subsp. aureus N315 SAS067	*mazEF*
Staphylococcus epidermidis ATCC 12228 SE1673	*mazEF*
Staphylococcus epidermidis RP62A SERP1682	*mazEF*
Streptococcus mutans UA159 SMU_172	*mazEF*
Synechococcus sp. WH 8102 SYNW2380	*mazEF*
Synechocystis sp. PCC 6803 ssl2245	*mazEF*
Thermoanaerobacter tengcongensis MB4	*mazEF*
Thermococcus kodakarensis KOD1 TK1815	*mazEF*
Brucella melitensis bv. 1 str. 16M BMEI1375	*phd-doc*
Brucella suis 1330 BR0558	*phd-doc*
Campylobacter jejuni RM1221 CJE1101	*phd-doc*
Candidatus Protochlamydia amoebophila UWE25 pc1457	*phd-doc*
Candidatus Protochlamydia amoebophila UWE25 pc1022	*phd-doc*
Enterococcus faecalis V583 EF0380	*phd-doc*
Methanosarcina mazei Go1 MM_0550	*phd-doc*
Mycobacterium smegmatis str. MC2 155 MSMEG_1277	*phd-doc*
Salmonella enterica subsp. enterica serovar Typhimurium str. LT2 STM3559	*phd-doc*
Streptococcus pneumoniae TIGR4 SP_0888	*phd-doc*
Streptomyces coelicolor A3(2) SCO5908	*phd-doc*
Xanthomonas axonopodis pv. citri str. 306 XAC1195	*phd-doc*
Staphylococcus aureus SACH_a19	*phd-doc*

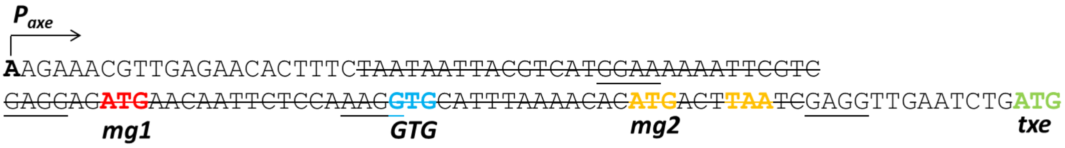

Figure S1. Nucleotide sequence of the *txe* leader where deleted fragment is indicated by a strikethrough line – used in the ATGtxe_w/o mg1,2 construct.

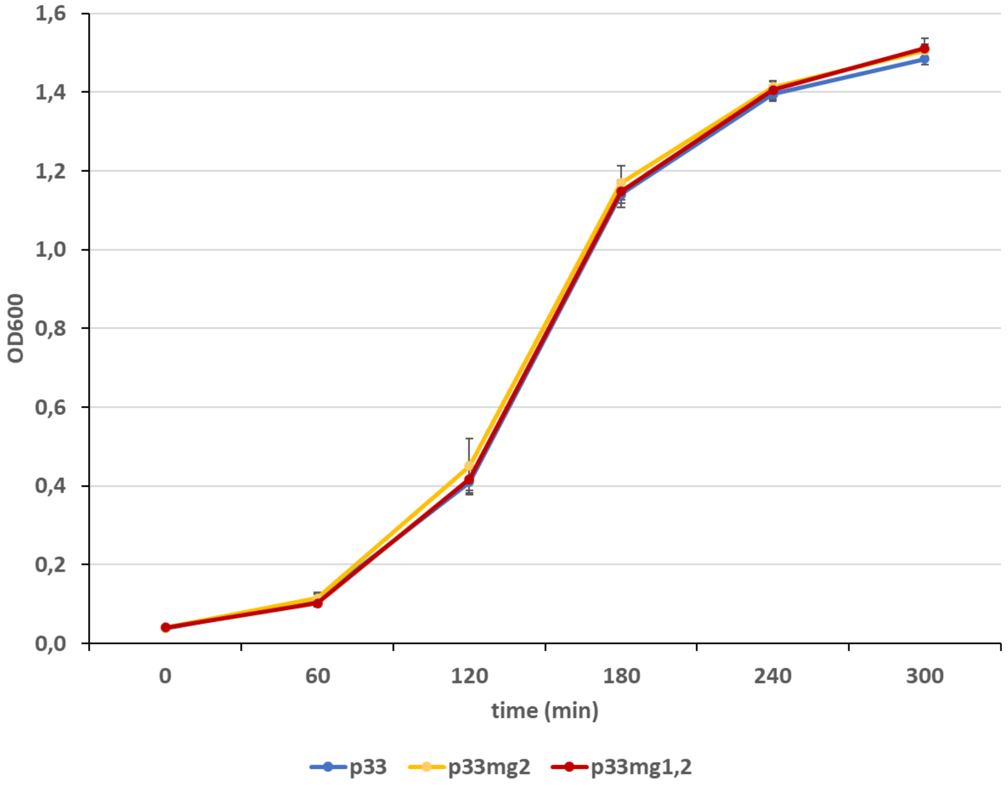

Figure S2. Minigenes encoded in the *txe* leader do not display toxic effect on bacterial cell growth. *E. coli* Δ5Δlac strain was transformed with pBAD33 or its derivatives, pBAD33_mg2 or pBAD33_mg1,2 bearing minigenes under *pBAD* promoter. Expression of minigenes was induced by addition of 0.2% L-arabinose at a time of inoculation. Absorbance readings at 600 nm were taken at 60 min. intervals. These results are average of five independent experiments, error bars represent standard deviation (S.D.).

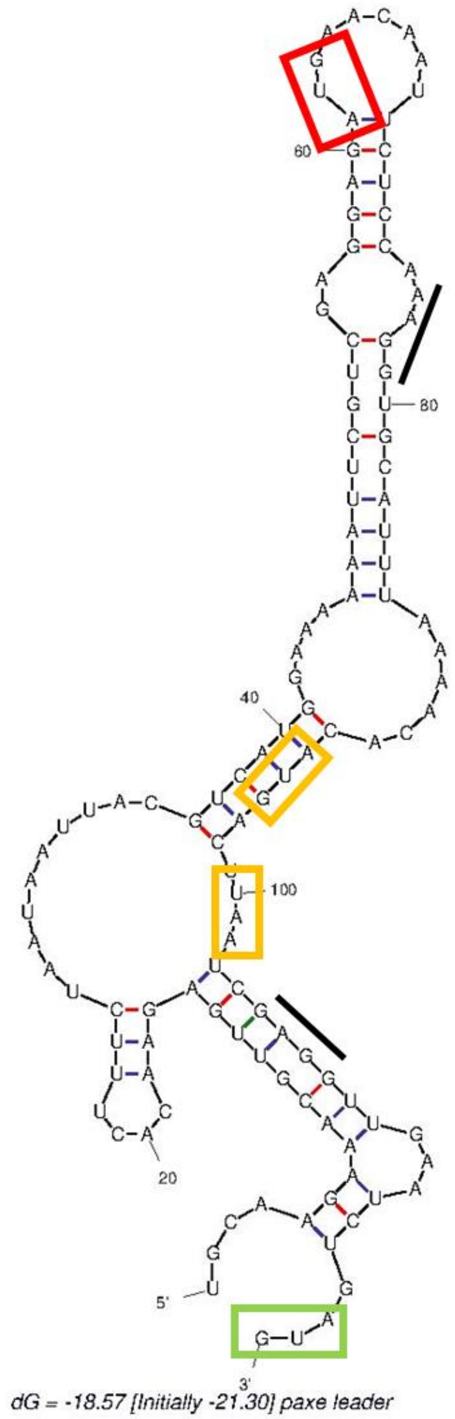

Figure S3. Secondary structure of *txe* leader fragment created with the mFOLD program (http://mfold.rna.albany.edu/). No evident strong secondary structures are formed. Start codons of *mg1*, *mg2* and ATGtxe are in colored boxes, red, yellow and green, respectively. UAA *mg2* stop codon is in yellow box. SD sequences are underlined.
